# Variation in zinc concentration of sweetcorn kernels reflects source–sink dynamics influenced by kernel number

**DOI:** 10.1093/jxb/eraa244

**Published:** 2020-05-22

**Authors:** Zhong Xiang Cheah, Tim J O’Hare, Stephen M Harper, Michael J Bell

**Affiliations:** 1 School of Agriculture and Food Sciences, The University of Queensland, Gatton, Queensland, Australia; 2 Queensland Alliance for Agriculture and Food Innovation, The University of Queensland, Gatton, Queensland, Australia; 3 Australia Department of Agriculture and Fisheries, Gatton, Queensland, Australia; 4 CSIRO Agriculture and Food, Australia

**Keywords:** Biofortification, grain, kernel mass, kernel number, maize, source–sink dynamics, sweetcorn, yield, *Zea mays*, zinc

## Abstract

Grain yield and mineral nutrient concentration in cereal crops are usually inversely correlated, undermining biofortification efforts. Here, sink size, expressed as kernel number per cob, was manipulated by controlling the time when the silks of sweetcorn (*Zea mays*) cv. Hybrix 5 and var. HiZeax 103146 were exposed to pollen. Twelve other varieties were manually pollinated to achieve the maximum potential kernel number per cob, and kernel Zn concentration was correlated with kernel number and kernel mass. As kernel number increased, kernel Zn concentration decreased, with the decrease occurring to similar extents in the embryo tissue and the rest of the kernel. However, total kernel Zn accumulated per cob increased with increasing kernel number, as the small decreases in individual kernel Zn concentration were more than offset by increases in kernel number. When both kernel number and mass were considered, 90% of the variation in kernel Zn concentration was accounted for. Differential distribution of assimilates and Zn to sweetcorn cobs led to significant decreases in kernel Zn concentration with increasing kernel number. This suggests there will be challenges to achieving high kernel Zn concentrations in modern high-yielding sweetcorn varieties unless genotypes with higher Zn translocation rates into kernels can be identified.

## Teaser

In sweetcorn, as kernel number increased, kernel Zn concentration decreased. However, total kernel Zn per cob increased, as the small decreases in kernel Zn concentration were offset by increases in kernel number.

## Introduction

Micronutrient deficiency is a severe nutritional problem affecting approximately 30% of the world’s population, mainly in developing countries (Kennedy *et al.*, 2003). One approach to addressing this issue of malnutrition has been through agronomic and genetic biofortification that aims to increase the concentration of a target micronutrient in the edible fraction of staple crops. Examples of species in which intensive biofortification efforts have been made include rice (*Oryza sativa*), wheat (*Triticum aestivum*), and maize (*Zea mays*) (White and Broadley, 2009). Despite these efforts, achieving the combination of high yield and high micronutrient concentration has proved challenging, as crop yield is usually inversely correlated to mineral nutrient concentration (Davis *et al.*, 2004; Murphy *et al.*, 2008). For example, decreases in the N, protein, and oil concentrations of shoots and kernels have been observed with increasing maize shoot biomass and kernel yield (Scott *et al.*, 2006; Riedell, 2010; Abdala *et al.*, 2018). Consequently, an assessment of kernel micronutrient concentration without consideration of yield may lead to inappropriate genotypic selections that do not provide successful biofortification outcomes.

An increase in grain crop yield may be due to an increase in kernel number or individual kernel mass (i.e. larger kernels) or both, with each parameter being influenced by genetic and environmental factors (Sadras, 2007). For modern maize hybrids, yield improvement has mostly been achieved through large increases in kernel number (Fischer and Palmer, 1984), which more than offsets the small decreases in individual kernel mass caused by limitations in the availability of assimilates (Echarte *et al.*, 2000). Varietal differences in kernel number and individual kernel mass between genotypes may also contribute to variation in kernel micronutrient concentrations (McDonald *et al.*, 2008). However, the evidence for these effects is not conclusive, with other studies finding the correlation between kernel Zn or Fe concentration and grain yield to be weak or non-significant (Ortiz-Monasterio and Graham, 2000; Long *et al.*, 2004; Chakraborti *et al.*, 2009). The opportunity for micronutrient biofortification in high-yielding maize varieties is therefore not well characterized.

Sweetcorn (*Zea mays* ssp. *saccharata*), which has a sugary endosperm conferred by a single gene mutation (Creech, 1965; Simonne *et al.*, 1999), is a close relative of maize. The micronutrients of importance to human health, such as Zn, are found in high concentrations in the scutellum of sweetcorn and maize embryos (Cheah *et al.*, 2019b). This micronutrient-rich embryo is typically removed during the processing of maize kernels by dry milling, whereas the entire kernel of sweetcorn is consumed. This potential dietary advantage of sweetcorn is negated by the fact that most of the Zn in the embryo is in the form of Zn phytate, which is not bioavailable to humans (Cheah *et al.*, 2019a); by contrast, the Zn accumulated in the endosperm has been shown to be mostly bioavailable. Therefore, to achieve a beneficial outcome for human health, genotypes that accumulate Zn in the endosperm are preferable to those that accumulate Zn in the embryo.

In previous experiments, we have observed that apparent genotypic differences in kernel Zn concentration were negatively correlated with kernel number per cob—that is, the net Zn supplied to a cob was diluted over a greater number of kernels. The aim of this study was to quantify the effect of sweetcorn kernel yield on kernel Zn concentration in a range of sweetcorn varieties. The relative changes in the accumulation and distribution of Zn between the embryo and the rest of the kernel with increasing kernel number were also evaluated. A better understanding of the relationship between kernel number and Zn accumulation will underpin successful selection strategies in breeding programmes that aim to develop sweetcorn varieties with high yield and greater Zn accumulation.

## Materials and methods

### Experimental setup

The experiments were conducted at the Gatton Research Facility of the Department of Agriculture and Fisheries (Gatton, Australia) on a Vertisol soil (Soil Survey Staff, 2014) with a DTPA-extractable Zn concentration of 4.6 mg kg^−1^. Fourteen sweetcorn varieties (listed in Table 3) were grown for this study at a density of 60 000 plants ha^−1^. Before planting, a basal fertilizer containing 55 kg N ha^−1^, 10 kg P ha^−1^, 55 kg K ha^−1^, and 80 kg S ha^−1^ was applied. Additionally, a urea–NH_4_–NO_3_ mix (Easy-N^®^) at a rate of 13.5 kg N ha^−1^, ammonium sulfate [(NH_4_)_2_SO_4_] at a rate of 125 kg ha^−1^, and magnesium sulfate (MgSO_4_) at a rate of 10 kg ha^−1^ were each applied once by drip irrigation. Finally, zinc oxide (ZnO) was applied once at the 12-leaf stage at a rate of 0.6 kg Zn ha^−1^ as a foliar application. During the growth period, the average minimum and maximum temperatures were 15 °C and 30 °C, respectively, and the relative humidity range was 44–59%. A completely randomized design was used for all experiments.

**Table 3.** Properties of 14 sweetcorn (*Zea mays*) varieties

Variety	Kernelnumber	Kernel DM (mg kernel^−1^)	Total kernel mass (g cob^−1^)	Kernel Zn concentration (mg kg^−1^ DM)	Total kernel Zn mass per cob (µg cob^−1^)	Zn partitioning capacity
O2su	458±16	46±6	21.07±0.10	29.8±3.2	627.9±0.32	Very good
HiZeax 103146	361±31	56±1	20.22±0.03	32.6±0.8	659.2±0.02	Very good
56.3–1	225±31	40±3	9.00±0.09	41.4±3.3	372.6±0.30	Moderate
14–6	146±47	34±5	4.96±0.24	43.9±6.4	217.7±1.54	Poor
Garrison (commercial)	487±14	57±3	27.76±0.04	19.9±1.0	552.4±0.04	Good
TF 2	432±18	37±3	15.98±0.05	23.7±1.2	378.7±0.06	Moderate
Hybrix 5 (commercial)	430±20	55±2	23.65±0.04	25.4±0.7	600.7±0.03	Very good
fl2sh2	360±36	26±6	9.36±0.22	24.9±1.9	233.1±0.42	Poor
EM 540	326±47	42±2	13.69±0.09	24.5±1.1	335.4±0.10	Moderate
6–1×15–2	311±33	62±5	19.28±0.17	28.3±1.1	545.6±0.19	Good
23–6	269±20	39±4	10.49±0.08	29.3±1.6	307.4±0.13	Moderate
23–1	230±43	51±3	11.73±0.13	33.2±2.6	389.4±0.34	Moderate
23–7	190±11	42±3	7.98±0.03	30.0±0.4	239.4±0.01	Poor
13–2	181±35	44±5	7.96±0.18	33.4±2.0	265.9±0.36	Poor

Data are expressed as means ± SE (*n* = 5 plants per variety). Classification of Zn partitioning capacity was added based on data of total kernel Zn mass per cob obtained in this study. The first four varieties listed expressed relatively higher kernel Zn concentration for the given kernel number (see Fig. 4). DM, Dry mass.

### Assessment of the accumulation of Zn and carbohydrate in the embryo and the rest of the kernel at various stages of maturity

The kernel number of the commercial variety cv. Hybrix 5 was manipulated by controlling the duration of exposure of the silks to pollen. Immediately before silk emergence, the ears of all plants were covered with a plastic bag. A visual assessment of peak pollen production (approximately 2 weeks after ear bagging) was used as a basis for treatment implementation. At this time, the bags were removed to expose the emerged silks to pollen for 0.5, 1, 2, 4, 8, 12, or 24 hours, and ears were subsequently covered with a paper bag to prevent further pollination. Ears were subsequently harvested at 18, 21, 24, and 28 days after pollination (DAP). The 21 and 24 DAP harvest times were equivalent to normal commercial sweetcorn harvest times, with the kernels at 18 DAP being slightly immature and those at 28 DAP being over-mature.

At harvest, cobs were categorized as well pollinated (>350 kernels), moderately pollinated (150–350 kernels), or poorly pollinated (<150 kernels). Five cobs were sampled from each category and 10 kernels were extracted from each cob. The kernels were dissected into embryo tissue and the rest of the kernel (including the endosperm, aleurone, and pericarp tissues), which were measured separately for tissue dry mass (DM) and Zn concentration. Kernel Zn content was then calculated as the sum of the products of the Zn concentration of each tissue (i.e. embryo or rest of the kernel) and DM content. The rates of kernel Zn and DM accumulation were calculated using the formula:

$$\begin{array}{l} R=\left(x_{end}-x_{start}\right)/\left(n\times d\right), \end{array}$$

where *x*_start_ and *x*_end_ represent the Zn or DM content at the start and end of a period, *n* represents the number of kernels, and *d* represents the number of days within that period.

### Assessment of the relationship between kernel number and Zn concentration within sweetcorn varieties

This experiment evaluated the relationship between kernel number and Zn concentration by comparing the sweetcorn varieties cv. Hybrix 5 and var. HiZeax 103146. Kernel number was manipulated using the method described above. Cobs were then harvested at 21 DAP, the kernel number was determined, and 10 kernels were extracted for nutrient analysis. Exposing silks to pollen for varying durations produced cobs with a wide variation in kernel number (range 1–554 kernels per cob). However, cobs with fewer than 10 kernels were excluded from the analysis due to an insufficient kernel mass for Zn determination. Total kernel Zn mass per cob was calculated using total kernel DM (g per cob) and Zn concentration (mg kg^−1^).

### Contribution of differences in kernel number and individual kernel mass to variation in kernel Zn concentration between sweetcorn varieties

The 14 sweetcorn varieties were manually self-pollinated to achieve the maximum potential kernel number. These varieties were harvested at 21 DAP (the normal harvest time for sweetcorn for human consumption), five cobs were sampled, and the kernel number per cob was determined. Ten kernels were extracted from each cob and their DM and mineral nutrient concentration were determined as described above. The relationships between kernel number, kernel DM, and kernel Zn concentration were determined.

### Mineral analysis

Kernels were removed from the cob for determination of fresh mass. Kernels were then oven-dried (70 °C) for determination of DM and tissue Zn concentrations. Dried kernel samples were digested in 6 ml nitric acid and 2 ml perchloric acid at 150 °C before being made up to 20 ml with deionized water. The digested samples were analysed using an inductively coupled plasma optical emission spectrometer (ICP-OES; Optima 7300 DV, Perkin Elmer; Wellesley, MA, USA) (Zasoski and Burau, 1977).

## Results

### Accumulation of dry matter and Zn during kernel development

**Fig. 1.**
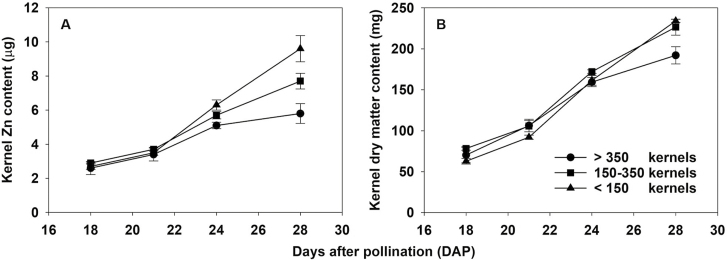
Accumulation of (A) Zn content and (B) dry matter content in individual kernels of sweetcorn (*Zea mays*) variety cv. Hybrix 5 at 18, 21, 24, and 28 days after pollination. Kernels were extracted from cobs that supported <150, 150–350, and >350 kernels.

The accumulation of Zn and dry matter in individual kernels of cobs of cv. Hybrix 5 with varying kernel numbers at different maturity stages (18–28 DAP) is shown in Fig. 1 and Table 1. The rate of accumulation of both Zn and dry matter was greatest at 21–24 DAP and was either maintained or declined to different extents at 24–28 DAP (Table 1). As kernel number increased, the relative changes in dry matter and Zn content were different. Specifically, the average Zn accumulation rate from 18 to 28 DAP was 0.7 µg Zn kernel^−1^ day^−1^ in poorly pollinated cobs, 0.5 µg kernel^−1^ day^−1^ in moderately pollinated cobs, and 0.3 µg kernel^−1^ day^−1^ in well pollinated cobs. Kernels on well pollinated cobs therefore accumulated Zn at *~*40% of the rate of kernels on poorly pollinated cobs. The average kernel dry matter accumulation rate decreased from 17.1 mg kernel^−1^ day^−1^ in poorly pollinated cobs (which had low kernel numbers) to 14.8 and 12.2 mg kernel^−1^ day^−1^ in moderately and well pollinated cobs, respectively. Kernels on well pollinated cobs therefore accumulated assimilate at ~70% of the rate of kernels on poorly-pollinated cobs—a much smaller reduction in accumulation than that recorded for kernel Zn.

**Table 1.** Accumulation of kernel Zn and dry matter content in sweetcorn (*Zea mays*) variety cv. Hybrix 5 between 18 and 28 days after pollination in poorly, moderately, or well pollinated cobs

Pollination success	Kernel number	Zn content (µg kernel^−1^ day^−1^)				Dry matter content (mg kernel^−1^ day^−1^)			
		18–21 DAP	21–24 DAP	24–28 DAP	Mean (18–28 DAP)	18–21 DAP	21–24 DAP	24–28 DAP	Mean (18–28 DAP)
Poorly pollinated	<150	0.3	0.9	0.8	0.7±0.2	9.8	23.1	18.2	17.1±3.9
Moderately pollinated	150–350	0.3	0.7	0.5	0.5±0.1	9.1	22.2	13.6	14.8±3.8
Well pollinated	>350	0.3	0.6	0.2	0.3±0.1	12.0	17.7	8.2	12.2±2.8

DAP, Days after pollination.

### Partitioning of Zn between the embryo and the rest of the kernel

**Fig. 2.**
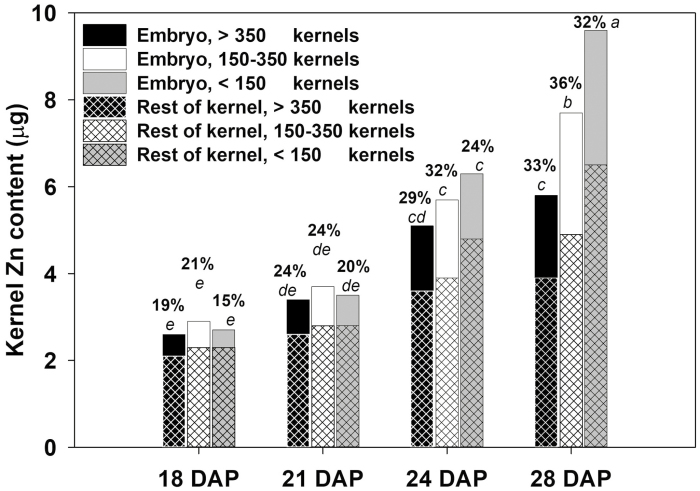
Accumulation of Zn content in the embryo and the rest of the kernel in individual kernels of sweetcorn (*Zea mays*) variety cv. Hybrix 5 at 18, 21, 24, and 28 days after pollination (DAP) for cobs supporting different numbers of established kernels. Percentage values show the proportion of whole kernel Zn content contained in embryo tissue. Different letters indicate significant differences (*P*<0.05) in whole kernel Zn content.

Given the marked difference in the relative accumulation of Zn and assimilate in kernels on cobs with different kernel numbers, the distribution of Zn between the embryo and the rest of the kernel was examined using cobs of cv. Hybrix 5 with varying kernel numbers. The Zn concentration in both embryo tissue and the rest of the kernel decreased by *~*18–33% as kernels matured over the period 18–28 DAP (Table 2). Despite the decrease in tissue Zn concentration, the Zn content of both the embryo and the rest of the kernel increased over the 18–28 DAP period as tissue DM increased (Fig. 2). The rest of the kernel constituted a much larger proportion of the kernel dry matter at all stages of kernel development (95% at 18 DAP, decreasing to 89% at 28 DAP). Hence, despite the much higher Zn concentration in embryo tissue (Table 2), the rest of the kernel contributed the majority of the whole kernel Zn content at all stages of maturity (Fig. 2). The proportion of the kernel Zn content contributed by the embryo doubled over the sampling period, from 15–21% at 18 DAP to 32–36% at 28 DAP, reflecting the relatively greater increase in embryo mass over the period. It is worth noting that kernel number had no real effect on the proportion of Zn content in the embryo and the rest of the kernel at any stage of kernel development. This similarity in response in both tissues indicated that in cobs with poor kernel establishment, the additional Zn available to each kernel was not preferentially stored in either tissue but was distributed in similar proportions across both tissues (Fig. 2).

**Table 2.** Zn concentration and dry mass of the embryo, rest of the kernel, and whole kernel in sweetcorn (*Zea mays*) variety cv. Hybrix 5 at 18, 21, 24, and 28 days after pollination in cobs with different kernel numbers

Tissue	Zn concentration (mg kg^−1^ DM)				Kernel dry mass (mg kernel^−1^)			
	18 DAP	21 DAP	24 DAP	28 DAP	18 DAP	21 DAP	24 DAP	28 DAP
**Embryo**								
>350 kernels	150±2.6 b,c	157±4.9 b	114±3.5 e	100±7.4 f	3±0.3 j	5±0.1 h	13±0.2 e	19±0.0 c
150–350 kernels	136±9.6 c,d	130±4.6 d	105±3.8 f	101±5.3 f	4±0.4 i	7±0.9 g	17±0.1 d	28±0.8 a
<150 kernels	167±4.2 a	135±7.1 c,d	142±6.7 c	134±2.5 c,d	2±0.4 k	5±0.2 h	11±0.6 f	23±0.2 b
**Rest of kernel**								
>350 kernels	31±1.6 b	26±1.4 d	25±0.9 d,e	23±1.5 e	67±4.0 f	101±6.2 d	146±5.2 c	173±6.1 b
150–350 kernels	31±1.6 b	28±0. 0c	25±0.7 d,e	25±1.8 d,e	74±2.0 e	99±5.8 d	155±4.1 c	199±7.5 a
<150 kernels	38±0.5 a	32±1.1 b	32±1.1 b	31±0.5 b	60±1.4 g	87±8.9 d	151±6.9 c	211±9.8 a
**Whole kernel**								
>350 kernels	37±1.2 c	32±2.9 d,e	32±0.7 d,e	30±2.0 d,e	70±3.1 h	106±8.3 e	159±6.4 d	192±5.1 b
150–350 kernels	37±1.6 b,c	35±1.1 c,d	33±1.6 d,e	34±2.0 c,d,e	78±2.4 g	106±7.2 e	172±2.7 c	227±6.1 a
<150 kernels	43±1.1 a	38±0.9 b,c	39±0.4 b	41±1.2 a	63±3.8 i	92±3.1 f	162±6.5 d	234±2.1 a

Within each type of tissue, means with different letters are significantly different (P<0.05). DAP, Days after pollination; DM, dry mass.

### Relationship between kernel number, kernel mass, and kernel Zn concentration

**Fig. 3.**
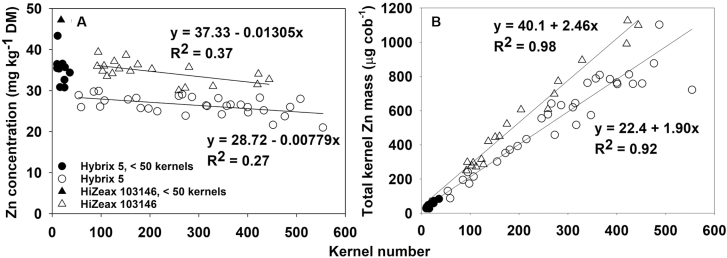
Relationships between sweetcorn (*Zea mays*) kernel number and (A) kernel Zn concentration on a dry mass (DM) basis and (B) total kernel Zn mass per cob in varieties cv. Hybrix 5 and var. HiZeax 103146 at 21 days after pollination.

The relationship between kernel Zn concentration and kernel number was similar for both cv. Hybrix 5 and var. HiZeax 103146, showing a consistent small decline (*P*=0.003) in kernel Zn concentration as kernel number increased from *~*50 kernels to the respective maximums in well pollinated cobs (*~*450 kernels in var. HiZeax 103146 and *~*550 kernels in cv. Hybrix 5; Fig. 3A). While the magnitude of the decline in Zn concentration with increasing kernel number appeared to be slightly greater for var. HiZeax 103146, the difference was not significant (*P*=0.261). There appeared to be a relatively sharp increase in kernel Zn concentrations at very low kernel numbers (below ~50 kernels) in cv. Hybrix 5, and although there was only one cob with a kernel number that low in var. HiZeax 103146, that sample also showed a similarly large increase in kernel Zn concentration. A significant varietal effect was also observed, with var. HiZeax 103146 having on average a 8.6±1.2 mg kg^−1^ (*P*<0.001) higher kernel Zn concentration than cv. Hybrix 5 at any given kernel number between *~*50 and ~550 kernels (Fig. 3A-a).

Owing to the statistically significant but relatively small decrease in kernel Zn concentrations with increasing kernel number for cobs with >50 kernels, total kernel Zn mass per cob for both varieties increased with increasing kernel number (Fig. 3B). The overall higher kernel Zn concentration resulted in a *~*30% higher (*P*=0.002) total kernel Zn mass per cob in var. HiZeax 103146 than in cv. Hybrix 5 in cobs with kernel numbers ranging from *~*200 to ~450 kernels.

### Relationships between kernel yield and Zn concentration in a collection of sweetcorn varieties

**Fig. 4.**
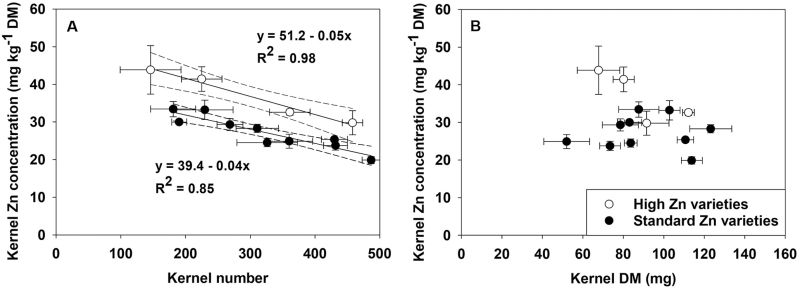
Relationships between kernel Zn concentration and (A) kernel number and (B) kernel dry mass for 14 sweetcorn (*Zea mays*) varieties grown in the field. Dashed lines in (A) show 95% confidence intervals.

The manually pollinated cobs from the 14 sweetcorn varieties produced different numbers of kernels, with different kernel mass and different kernel Zn concentrations (Table 3). A visual assessment of cobs showed that the pollination effectiveness (the proportion of total cob length hosting kernels) ranged from 70% to 100%. Across all 14 varieties, there was a strong correlation between kernel Zn concentration and kernel number (*R*^2^=0.57, *P*=0.002; Fig. 4A), but there was no correlation between kernel DM and Zn concentration (*R*^2^=0.05, *P*=0.450; Fig. 4B), suggesting that kernel number was the more important factor in determining genotypic differences in kernel Zn concentration. There appeared to be a subset of four genotypes in which higher Zn concentrations were recorded than would be expected from the kernel numbers present, with a separate relationship for kernel number and kernel Zn concentration established for this subset (Fig. 4A). These genotypes had kernel Zn concentrations that were 11.9±2.9 mg Zn kg^−1^ higher (*P*=0.002) for any given kernel number than the broader population, but the combination of low kernel numbers and low kernel DM resulted in two of these genotypes (56.3–1 and 14–6) having only a low to moderate total kernel Zn mass per cob (Table 3).

**Fig. 5.**
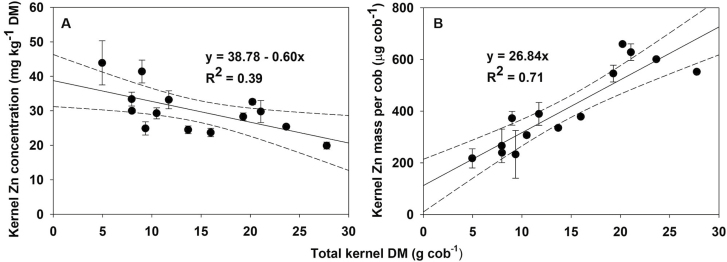
Relationships between total kernel dry mass (DM) per cob and (A) kernel Zn concentration and (B) total kernel Zn mass per cob and for 14 sweetcorn varieties. Data are presented as means with error bars indicating SE (*n* = 5 plants per variety).

The relationship between yield and kernel Zn was explored, using total kernel DM per cob (i.e. the product of kernel number and kernel DM) as a proxy for yield. As expected, there was a negative correlation between total kernel DM and kernel Zn concentration (*P*=0.017; Fig. 5A). However, total kernel DM was positively correlated with total kernel Zn mass accumulated per cob (*P*<0.001; Fig. 5B), with the variability in this relationship representing a measure of differences in the capacity of different varieties to translocate Zn into kernels. Multiple linear regression analysis showed that most of the variability in the total kernel Zn mass per cob (*R*^2^=0.93, *P*<0.001) across these genotypes could be accounted for by differences in kernel number per cob and individual kernel Zn concentration. Different genotypes were therefore able to be ranked in terms of their ability to partition Zn into developing kernels (Table 3).

## Discussion

### Effects of variability in yield influenced by source–sink dynamics on kernel Zn concentration

The dilution of mineral nutrient concentrations by high biomass production or grain yield is a well reported phenomenon in crops (Jarrell and Beverly, 1981; Davis, 2009; Riedell, 2010). However, no studies have explored the physiological mechanisms underpinning the negative correlation between micronutrient concentrations and grain yield. In this study, we examined the relationship between kernel Zn concentration and the two components of grain yield, namely kernel number and kernel mass, in sweetcorn. Both of these factors are important for yield improvement, as changes in kernel number may be compensated for by changes in kernel mass and vice versa (Sadras, 2007).

We demonstrated that as the sink demand for assimilates increased due to increasing numbers of established kernels, the relative change in the rate of accumulation of carbohydrates in kernels was relatively less affected compared with that of kernel Zn (Fig. 1, Table 1). The suggestion that the distribution of assimilates to developing kernels was not strongly source-limited was consistent with the accumulation behaviour of assimilates in maize, where assimilate accumulation was found to be sink-limited in most growing conditions and source-limited only if assimilate availability was reduced during grain filling due to poor growing conditions (Echarte *et al.*, 2000; Borrás *et al.*, 2004).

The greater sensitivity of Zn accumulation to increasing sink size (due to higher kernel numbers) was manifested in declining individual kernel Zn concentrations with increasing kernel numbers, and was indicative of source limitations that will constrain Zn accumulation in kernels of sweetcorn. This relationship was explored in two varieties: a commercially cultivated variety, cv. Hybrix 5, and an experimental variety previously identified for its higher kernel Zn concentration, var. HiZeax 103146. We found that the same negative correlation between kernel Zn concentration and kernel number existed in both varieties (Fig. 3A), indicating that it is possible that observed differences in kernel Zn concentration between varieties could be strongly influenced by the number of kernels set. Indeed, the negative correlation between kernel Zn concentration and kernel number explained a significant proportion of the variation in kernel Zn concentration across a broader population of 14 genotypes (Fig. 4A).

While the relationship between kernel number (one of the main contributors to yield increases in sweetcorn breeding) and kernel Zn concentration was observed to hold across a set of 14 genotypes, the relationship between kernel mass (the other key yield key determinant in sweetcorn) and kernel Zn concentration was weak (Fig. 4b). This suggests that the decreases in kernel Zn concentration that have been observed with increasing sweetcorn yields are likely to be mainly driven by increases in kernel number rather than kernel mass. Kernel mass in maize was reported to be strongly influenced only by reduction in potential assimilate availability leading to reduced kernel mass, but increases in potential assimilate availability did not result in improved kernel mass (Borrás *et al.*, 2004). Conversely, the selection pressure for higher yields in maize, achieved mainly through increased kernel numbers (Fischer and Palmer, 1984), could have unintentionally selected for lower kernel Zn concentration in modern maize hybrids.

While the individual kernel Zn concentration decreased with increasing yield, the total amount of Zn being translocated into the kernels increased with yield, maximizing the overall total kernel Zn mass per cob (Fig. 5B). The small decrease in individual kernel Zn concentration associated with increases in kernel number was more than offset by the increase in kernel mass per cob, and this observation suggests that the effect of source limitations in constraining kernel Zn concentration is relatively small (Fig. 3b). This implies that while both individual kernel Zn concentration and total kernel yield are valid selection parameters, the most efficient way of increasing total Zn yield ha^−1^ in the edible product is by increasing sweetcorn yield, regardless of whether that increase is due to higher kernel numbers, higher kernel DM, or both. Future studies could explore whether the negative correlation between kernel number and Zn concentration applies only to a single cob or to multiple cobs on a plant. If the former were true, there could be potential to increase kernel Zn concentrations while maintaining or improving yield by selecting for genotypes with multiple smaller cobs that each support fewer kernels.

### Breeding strategies to improve dietary Zn intake from sweetcorn

From the dietary perspective, there are two different breeding strategies to achieve biofortification outcomes in high-yielding sweetcorn varieties, depending on the target market and consumption pattern. If catering to markets that consume whole cobs, selection for any combination of increased kernel number and/or kernel mass will increase the total kernel Zn mass per cob, and hence dietary intake. However, in markets where cut ‘cobettes’ or processed kernels in fixed-weight packaging are preferred, the dietary intake will be based on kernel concentration, so higher yields will have to be achieved through greater kernel mass to avoid any negative impacts of increased kernel numbers on kernel Zn concentration. Given the dominance of increased kernel number driving yield increases in modern maize hybrids (Fischer and Palmer, 1984) and the apparently strong impacts of the environment on the stability of genotypic differences in individual kernel mass, the latter strategy presents significant challenges.

On a positive note, var. HiZeax 103146 exhibited higher kernel Zn concentrations than cv. Hybrix 5 at any given kernel number (Fig. 3A). This finding suggested that there are genotypes that are more efficient at translocating Zn into cobs for distribution across the developing kernels, a trait that is independent of kernel number variation, which was also observable in a subset of the 14 genotypes studied (Fig. 4A). Although the mechanisms for efficient uptake and accumulation of Zn in these varieties are unclear and warrant further investigation, these traits offer valuable resources to be exploited for genetic biofortification of Zn in sweetcorn. Any genetic advances will need to be supported by appropriate agronomic biofortification strategies to achieve the desired enhancement of Zn partitioning and the concentration of Zn in individual kernels.

### Accumulation of Zn and assimilates in the embryo and the rest of the kernel

Given the importance of speciation to the bioavailability of Zn for absorption after consumption, the allocation of Zn between embryo and endosperm tissues will be an important factor in securing desired biofortification outcomes. This is important because of the contrasting Zn concentrations (Table 2) in these kernel constituents and their bioavailability: Zn in embryo tissues is stored predominantly as Zn phytate, which has low bioavailability for humans, whereas Zn stored in the endosperm is complexed with N- or S-containing ligands and has higher bioavailability (Cheah *et al.*, 2019a). In addition, the stability of the Zn allocation between kernel constituents in response to increasing source limitations caused by increasing kernel number will be particularly important for breeding programmes seeking to improve both yield and bioavailable Zn content in sweetcorn.

In this study, the trends in Zn concentration and Zn content of embryo tissue and the rest of the kernel as the kernels mature on a well pollinated cob of sweetcorn were similar to those observed in earlier studies (Cheah *et al.*, 2020). We showed that the ratio of Zn content in the embryo to Zn content in the rest of the kernel was constant irrespective of the established kernel number, suggesting that there was no preferential storage of Zn in either tissue with increasingly constrained Zn supplies. This observation implies that in order to further improve sweetcorn as a source of dietary Zn, there may be scope to change the relative proportions of Zn in different kernel constituents by reducing the mass and/or volume ratio of the embryo relative to the endosperm or the rest of the kernel (Zhang *et al.*, 2012; Nagasawa *et al.*, 2013; Chen *et al.*, 2014; Golan *et al.*, 2015; Suzuki *et al.*, 2015).

## Conclusion

As the number of established kernels on a sweetcorn cob increased, plants were better able to maintain the rate of assimilate supply than the rate of Zn supply to the developing kernels. This resulted in a decrease in kernel Zn concentration as the kernel number increased. However, increases in kernel mass per cob (the product of kernel number and DM content) due to increased kernel number more than offset the small decreases in individual kernel Zn concentrations, such that the total kernel Zn mass per cob was maximized when grain yield was maximized. This result suggests that in the absence of sink size limitations, increasing the supply of Zn into the kernels, either through enhanced genotypic efficiency in translocating Zn or by ensuring adequate Zn availability via agronomic means, would be needed to maintain high kernel Zn concentrations. Targeted crosses of high-yielding varieties with efficient kernel Zn accumulation could potentially achieve concomitant improvements in both Zn concentration and yield in modern sweetcorn cultivars.

## Abbreviations

DAP: days after pollination
DM: dry mass
